# Sirtuins as Potential Targets for Neuroprotection: Mechanisms of Early Brain Injury Induced by Subarachnoid Hemorrhage

**DOI:** 10.1007/s12975-023-01191-z

**Published:** 2023-10-02

**Authors:** Kunqian Lei, Rui Wu, Jin Wang, Xianze Lei, Erxiong Zhou, Ruiming Fan, Lei Gong

**Affiliations:** 1 Department of Neurosurgery, Affiliated Hospital of Zunyi Medical University CN, Zunyi, China; 2 Department of Neurology, Affiliated Hospital of Zunyi Medical University CN, Zunyi, China; 3 Department of Pharmacy, Institute of Medical Biotechnology, Affiliated Hospital of Zunyi Medical University CN, Zunyi, China

**Keywords:** Subarachnoid hemorrhage, Early brain injury, Sirtuins (SIRTs), Mechanism, Mitochondrial dysfunction, Ferroptosis,apoptosis, Autophagy,neuroinflammation, Oxidative stress, Blood–brain barrier

## Abstract

Subarachnoid hemorrhage (SAH) is a prevalent cerebrovascular disease with significant global mortality and morbidity rates. Despite advancements in pharmacological and surgical approaches, the quality of life for SAH survivors has not shown substantial improvement. Traditionally, vasospasm has been considered a primary contributor to death and disability following SAH, but anti-vasospastic therapies have not demonstrated significant benefits for SAH patients' prognosis. Emerging studies suggest that early brain injury (EBI) may play a crucial role in influencing SAH prognosis. Sirtuins (SIRTs), a group of NAD + -dependent deacylases comprising seven mammalian family members (SIRT1 to SIRT7), have been found to be involved in neural tissue development, plasticity, and aging. They also exhibit vital functions in various central nervous system (CNS) processes, including cognition, pain perception, mood, behavior, sleep, and circadian rhythms. Extensive research has uncovered the multifaceted roles of SIRTs in CNS disorders, offering insights into potential markers for pathological processes and promising therapeutic targets (such as SIRT1 activators and SIRT2 inhibitors). In this article, we provide an overview of recent research progress on the application of SIRTs in subarachnoid hemorrhage and explore their underlying mechanisms of action.

## Introduction

Subarachnoid hemorrhage (SAH) is a prevalent cerebrovascular disease with varying incidence rates across different countries and regions. Globally, the overall incidence is estimated to be around 6 per 100,000 individuals annually [1]. The occurrence of subarachnoid hemorrhage tends to increase with age. Despite notable advancements in surgical interventions such as aneurysm clamping and endovascular occlusion, the long-term prognosis for SAH patients remains suboptimal. Delayed cerebral ischemia (DCI), which typically manifests 3–14 days after SAH, has long been recognized as a significant factor contributing to the poor prognosis of SAH patients [2].

Historically, cerebral vasospasm (CVS) has been regarded as the primary cause of DCI following SAH. Consequently, many studies over the past decades have focused on reducing episodes of vasospasm to improve the prognosis of SAH patients [3–7]. However, recent large-scale clinical trials have revealed that treating vasospasm does not yield substantial improvements in patient outcomes [8–10]. This suggests that other mechanisms of injury may play a role in determining the prognosis of SAH patients. An increasing body of evidence highlights the importance of early brain injury (EBI), which refers to brain damage occurring within 72 h after subarachnoid hemorrhage [11].

Recent findings indicate that EBI after SAH may lay the foundation for the subsequent development of DCI, characterized by complex pathophysiological changes such as increased intracranial pressure, reduced cerebral blood flow, and direct hematoma toxicity in brain tissue. These processes are followed by blood–brain barrier (BBB) disruption, oxidative stress injury, cell death, inflammatory response, microcirculatory dysfunction, and mitochondrial dysfunction, all contributing to neurological damage and poor prognosis after SAH [12–16].

In recent years, there has been a growing interest in the early diagnosis and treatment of SAH, focusing on initiating neuroprotective measures and early prevention of complications during the initial bleeding stages. This approach aims to mitigate the impact of EBI and improve patient outcomes.

Sirtuins (SIRTs) are a class of NAD + -dependent protein lysine deacylases and ADP ribosylases present in both prokaryotes and eukaryotes. Within the family of histone deacetylases (HDACs), SIRTs are classified as class III HDACs, acting in opposition to the activity of histone acetyltransferases (HATs) [17, 18, 19]. Currently, there are seven known isoforms of sirtuins (SIRT1-7) that exhibit distinct subcellular localizations and substrate specificities. SIRT1, SIRT6, and SIRT7 are primarily located in the nucleus, although a fraction of SIRT1 is also found in the cytoplasm. Conversely, SIRT2 is predominantly cytoplasmic, but certain splice isoforms can be present in the nucleus under specific conditions. SIRT3-5 are primarily localized within mitochondria [20].

Sirtuins play crucial roles in various biological processes, including cell differentiation, transcriptional regulation, cell cycle progression, apoptosis, inflammation, metabolism, as well as neurological and cardiovascular physiology. Over the past two decades, there has been a growing interest in exploring the functions of sirtuins, and they have garnered significant attention [21]. In recent years, the role and mechanisms of sirtuins in SAH have been extensively investigated. The findings suggest that sirtuins are involved in multiple mechanisms that alleviate brain injury following SAH, thereby reducing neurological impairment and improving prognosis. Consequently, modulating sirtuin activity has emerged as a promising therapeutic approach. This review aims to summarize and discuss the existing literature on the involvement of sirtuins in EBI after SAH. The goal is to elucidate the underlying mechanisms responsible for inducing neuroprotection and provide a theoretical basis for understanding the mechanisms and treatment options for EBI following SAH.

## The Role and Mechanism of Sirtuins in Subarachnoid Hemorrhage

### Sirtuins and Mitochondrial Dysfunction

Mitochondria, double-membrane organelles, are pivotal in energy production within eukaryotic cells. They play crucial roles in cell growth, differentiation, function, signal transduction, cell cycle regulation, apoptosis, and survival [22–24]. Impaired mitochondrial function can have detrimental effects, including the loss of mitochondrial membrane potential (ΔΨm), excessive reactive oxygen species (ROS) production, release of apoptogenic proteins, compromised mitochondrial dynamics, and activation of inflammation associated with mitochondria [25, 26]. Recent evidence highlights mitochondrial dysfunction as a novel mechanism and target for EBI in relation to delayed cerebral ischemia (DCI) and SAH outcomes [27]. Additionally, mitochondrial autophagy, known as mitophagy, is activated following SAH and may act as a neuroprotective mechanism [28].

Mitochondrial dynamics, which involve fusion and division processes, have gained considerable attention due to their involvement in various biological phenomena such as apoptosis, senescence, and mitochondrial autophagy [29, 30]. Mitochondrial fusion relies on three guanosine triphosphate (GTP) hydrolases: mitochondrial fusion proteins (Mfn) 1 and 2 facilitate outer membrane fusion, while optic atrophy-associated protein 1 (OPA-1) mediates inner membrane fusion [31]. Under normal circumstances, mitochondrial fusion maintains organelle integrity through the diffusion and sharing of components [32]. Animal studies have demonstrated a significant decrease in the expression of Mfn1 and Mfn2 in the central nervous system (CNS) 24 h after SAH, while OPA-1 expression decreased starting from 3 h post-SAH and continued to decline for up to 72 h [33, 34]. Interestingly, both studies revealed that increased expression of Mfn1/2 and OPA-1 facilitated the mitigation of EBI following SAH [33, 34]. Mitochondrial autophagy, a selective process responsible for removing damaged mitochondria, promotes mitochondrial homeostasis. Accumulation of impaired mitochondria can increase oxygen consumption and ROS production, leading to cellular degeneration and activation of cell death pathways [35]. Abundant evidence supports mitochondrial dysfunction after SAH and its association with EBI and neurological outcomes [36–39]. Therefore, further investigations are warranted to elucidate the underlying mechanisms of mitochondrial dysfunction in the development of EBI after SAH (Table 1).

**Table 1:** (continued) Summary of the most relevant in vivo preclinical studies evaluating the role of SIRTs in experimental animal models of SAH

Activators or inhibitors of SIRTs	In vivo or in vitro models of SAH	Types of mice	Participation mechanisms	Influence	References
Activator: Honokiol (HKL)	Endovascular perforation method	Male C57BL/6 mice	Activation of the SIRT3/ AMPK pathway upregulated the expression of Mfn1 and Mfn2	Protects mitochondrial morphology and promotes neuronal survival	Wu et al. [33]
-	Endovascular perforation method	male Sprague–Dawley rats	Activation of MC1R via the AMPK/SIRT 1/PGC-1α pathway	Inhibition of oxidative stress, apoptosis and mitochondrial fission	Xu et al. [40]
Activator: Fucoxanthin (Fx) Inhibitor: EX527	Autologous blood injection method and in vitro models	Adult male Sprague–Dawley rats and pregnant C57BL/6 mice	activates Sirt1, inhibits acetylation of FoxO1 and p53, and blocks mitochondrial release of cyt c	Restore mitochondrial morphology and improve oxidative damage	Zhang et al. [41]
-	Autologous blood injection method and in vitro models	male Sprague–Dawley rats	Inhibition of Mfn1-IIPKC signalling activates SIRT3 and its mitochondrial antioxidant enzyme activity	Attenuates neuronal damage and mitochondrial dysfunction	Chen al. [44]
Activator: Resveratrol (RVS)	Endovascular perforation method	Male mice (C57BL/6 wild type [WT])	Activation of Sirt5 to disrupt lysine succinylation	Restoration of mitochondrial metabolism and amelioration of neuronal cell death	Xiao et al. [45]
Activator: resveratrol (RVS) Inhibitor:selisistat (SEL)	Autologous blood injection method and in vitro models	Male C57BL/6 mice	SIRT1 activation up-regulates the expression of GPX4 and FSP1	Reduces lipid peroxide levels and alleviates ferroptosis	Yuan et al. [57]
Activator: activator 3 Inhibitor: sirtinol	Autologous blood injection method	Male Sprague–Dawley rats	SIRT1 deacetylation inhibits FoxOs, NF- kB and p53 expression	Inhibits oxidative stress, inflammation and apoptosis pathways	Zhang et al. [75]
Activator: Cycloastragenol (CAG) Inhibitor: Ex527	Endovascular perforation method and in vitro models	Adult male Sprague–Dawley rats	SIRT1 expression was upregulated, microglia activation was inhibited, and FoxO1, NF-kB and p53 acetylation levels were suppressed	Inhibits oxidative, inflammatory and apoptotic pathways	Lin et al. [76]
Activator: Magnesium Lithospermate B (MLB) Inhibitor: sirtinol	Endovascular perforation method	Adult male Sprague–Dawley rats	activation of SIRT1, inhibition of NF-κB acetylation and downregulation of cleaved caspase-3 expression	Inhibits inflammation and apoptosis	Peng et al. [77]
Activator: Astaxanthin (ATX) Inhibitor: sirtinol	Autologous blood injection method and in vitro models	Adult male Sprague–Dawley rats, wild-type (WT)C57BL/6 mice and TLR4 gene knockout (KO) mice	activation of SIRT1 decreased the level of HMGB1 and reduced the nuclear translocation of TLR4	Reduces brain inflammation and improves neurological function	Zhang et al. [78]
Activator: Pinocembrin Inhibitor: Ex527	Endovascular perforation method	male Sprague–Dawley rats	Enhanced expression of SIRT1 and Pgc1-α and decreased expression of ac-NF-κb	Inhibits inflammation, free radical damage and neuronal damage	Zeng et al. [79]
Activator: Resveratrol (RVS)	Endovascular perforation method	Male Sprague–Dawley rats	Elevated phosphorylation status of AMPK, SIRT1 protein expression	Activation of autophagic pathway attenuates inflammatory damage and apoptosis	Li and Han [80]
Activator: Melatonin(MEL) Inhibitor: Sirt1 siRNA	Endovascular perforation method	adult male C57BL/6 J mice	Increased Sirt1, Bcl-2 expression, decreased Ac-NF-κB, Bax expression	Reduces brain edema and apoptosis	Zhao et al. [81]
Activator: Rolipram	Endovascular perforation method	Adult male Sprague–Dawley rats	SIRT1 activation, inhibition of NF-κB activation and microglial cell activity	Inhibits inflammation and neuronal damage	Peng et al. [82]
Activator: Berberine Inhibitor: EX527	Autologous blood injection method	Adult male Sprague–Dawley rats	Activation of SIRT1 inhibits HMGB1/Nf-kB signaling pathway	Reducing brain inflammation	Zhang et al. [84]
Activator: Oleanolic acid (OA) Inhibitor: sirtinol	Endovascular perforation method	Adult male Sprague–Dawley rats	activates SIRT1, enhances the deacetylation of HMGB1, and reduces the serum HMGB1	Inhibits inflammation and reduces neuronal apoptosis	Han et al. [85]
Activator: Dioscin Inhibitor: Ex527	Autologous blood injection method	Adult male Sprague–Dawley rats and pregnant C57BL/6 mice	Activates SIRT1, inhibits NLRP3 inflammatory vesicles, and reduces ROS production	Improves cellular viability, inflammation, oxidative stress and neuronal damage	Zhang et al. [87]
Activator: SRT1720 Inhibitor: EX527	Autologous blood injection method	male Sprague–Dawley rats	Activation of SIRT1 decreases FOXO1 acetylation and inhibits NLRP3 inflammatory vesicle signaling	Polarization shift of microglia from M1 phenotype to M2 phenotype	Xia et al. [88]
Activator: Estrogen	-	female Sprague Dawley rats	Estrogen deficiency leads to downregulation of ERα and Sirt1 and an increase in NLRP3, IL-1β and MMP-9	Facilitating the rupture of IAs in the estrogen-deficient rat IA rupture model	Yamaguchi et al. [89]
Inhibitor: OSS128167	Endovascular perforation method	male Sprague–Dawley rats	activation of RXR, SIRT6 and PPARγ and decreased FoxO3a phosphorylation and pro-inflammatory cytokine expression	Improve nerve inflammation	Zuo et al. [90]
Activator: Salvianolic acid B (SalB) Inhibitor: sirtinol	Autologous blood injection method and in vitro models	male C57BL/6 (wild-type, WT) mice	Activation of SIRT1 induces Nrf2 nuclear translocation and inhibits ROS production	Inhibits oxidative damage and reduces neuronal degeneration	Zhang et al. [104]
Activator: Isoliquiritigenin (ISL) Inhibitor: EX527	Autologous blood injection method and in vitro models	Adult male Sprague–Dawley rats and neonatal C57BL/6 mice	SIRT1 regulates Nrf2-dependent antioxidant pathways, including HO-1, NADPH:NQO-1, and SOD	Improve oxidative damage	Liu et al. [105]
Activator: pterostilbene (PTE) Inhibitor: EX527	Autologous blood injection method	Adult male Sprague–Dawley rats	Enhanced expression of SIRT1 and antioxidant enzymes, inhibition of Nrf2 and microglia activation	Reduces inflammatory response, oxidative damage and neuronal death	Zhang et al. [106]
-	Autologous blood injection method and in vitro models	Male C57BL/6 mice	Increased expression of PGC-1a and SIRT3 in neurons in vivo and in vitro	Prevention of oxidative stress	Zhang et al. [108]
-	Endovascular perforation method	Adult male Sprague–Dawley rats	SOD2 expression was positively correlated with the rise in SIRT3	Prevention of oxidative stress	Huang et al. [109]
Activator: wogonoside	Endovascular perforation method	male Sprague–Dawley rats	Activation of SIRT1, upregulation of ZO-1, Occludin and Claudin-5 proteins and downregulation of p53	Blocking brain edema progression and neuronal cell apoptosis	Cheng et al. [136]
Activator: Resveratrol (RVS) Inhibitor: Sirtinol	Endovascular perforation method	Adult male Sprague–Dawley rats	Activation of SIRT1, upregulation of ZO -1, Occludin and Claudin -5, downregulation of p53 and apoptosis proteins	Improved brain edema and reduced apoptosis due to blood–brain barrier disruption	Qian et al. [137]
Inhibitor: sirtinol	Endovascular perforation method	Adult male Sprague–Dawley rats	SIRT1 promotes phosphorylation and activation of Akt leading to an increase in the Bcl-2/Bax ratio	Reduced brain edema and neuronal cell apoptosis	Li et al. [138]
Activator: Puerarin (Pur)	Endovascular perforation method	adult male C57BL/6 J mice	Activates SIRT3, downregulates SOD2 and MDA, and inhibits the Bax/Bcl-2/cleaved caspase-3 apoptotic signaling pathway	Improved oxidative stress and apoptosis	Zhang et al. [139]
Activator:Tauroursodeoxycholic acid (TUDCA)	Endovascular perforation method	Adult male Sprague–Dawley rats	Upregulation of SIRT3 and BCL-2 decreased the expression of BAX and cleaved caspase3	Attenuates apoptosis	Wu et al. [140]
Inhibitor: sirtinol	Autologous blood injection method	Adult male Sprague–Dawley rats	endogenous SIRT1 was reduced, leading to increased levels of p53 and MMP-9	Increase in BBB permeability and brain edema	Zhou et al. [160]

"-" stands for nil

Sirtuins have been implicated in the regulation of mitochondrial function in SAH. Activation of the MC1R by BMS-470539 has been shown to attenuate EBI after SAH by promoting mitochondrial biogenesis and controlling mitochondrial metabolism through the AMP-activated protein kinase (AMPK)/SIRT1/PGC-1α pathway [40]. Additionally, fucoxanthin (FX), a derivative of luteolin, restores mitochondrial morphology by activating SIRT1 and preventing cytochrome c release from mitochondria [41].

SIRT3 is predominantly localized in the mitochondrial matrix and plays a crucial role in regulating mitochondrial metabolism, the tricarboxylic acid (TCA) cycle, urea cycle, amino acid metabolism, fatty acid oxidation, electron transport chain (ETC)/oxidative phosphorylation (OXPHOS), detoxification of reactive oxygen species, mitochondrial dynamics, and mitochondrial unfolded protein response (UPR) [42, 43]. SIRT3 improves EBI after SAH by promoting mitochondrial fusion in an AMPK-dependent manner. Activation of SIRT3 by Honikiol (HKL) increases the levels of mitochondrial fusion proteins Mfn1 and Mfn2, thereby maintaining mitochondrial morphology, protecting mitochondrial function, and promoting neuronal cell survival [33].

Moreover, IIPKC is an isoform of protein kinase C (PKC) that interacts with Mfn1 to mediate mitochondrial dysfunction and neuronal damage after SAH, both in vitro and in vivo. The interaction between Mfn1 and IIPKC can be blocked by activating the SIRT3 pathway, as observed in experiments where Sirt3 was knocked down with small interfering RNA (siRNA) [44]. Additionally, protein lysine succinylation serves as a biochemical marker of metabolic crisis after SAH. In animal studies of SAH, resveratrol (RVS) activation of SIRT5-mediated blockade of lysine desuccinylation protected mitochondrial metabolism after SAH, ameliorating neuronal cell death and neurological deficits [45].

Therapies targeting mitochondria in the context of SAH have shown potential in animal studies. The regulation of mitochondrial function by SIRTs offers new perspectives for future studies on the mechanisms and treatment of EBI after SAH.

### Sirtuins and Ferroptosis

Ferroptosis, a novel form of cell death first defined in 2012, is characterized by non-apoptotic cell demise, iron dependency, and accumulation of reactive lipid substances (RLS) [46]. Ferroptosis exhibits distinct morphological and bioenergetic features that set it apart from other established forms of regulated cell death. Morphologically, it primarily affects mitochondria, leading to smaller mitochondria, increased mitochondrial membrane density, disrupted or absent mitochondrial cristae, and damaged outer mitochondrial membranes, while the nuclear morphology remains unaffected. Bioenergetically, ferroptosis is characterized by iron accumulation and lipid peroxidation [47].

The regulatory mechanism of ferroptosis is complex and still being extensively studied. Current research suggests that the regulation of iron, lipid peroxidation, and various antioxidant systems, such as the GSH/GPX4 pathway, CoQ10/FSP1 pathway, and others, play crucial roles in the regulation of ferroptosis [48]. Iron's involvement in ferroptosis primarily occurs through the Fenton reaction, which catalyzes the generation of ROS and ultimately leads to cell death [49]. Lipid peroxidation and subsequent accumulation of oxidized products are hallmark features of ferroptosis [50]. However, it should be noted that only the esterification of phospholipids into the membrane can induce ferroptosis, and lipid peroxidation can affect various cellular membranes, including the lipid bilayer and subcellular membranes of mitochondria, endoplasmic reticulum, and lysosomes [51].

Iron-induced apoptosis has been implicated in the pathology of EBI after SAH. Notably, ferroptosis is not an isolated pathological process but rather shares commonalities with other forms of cell death, including apoptosis, necroptosis, necrosis, and autophagy [52–54]. Furthermore, ferroptosis exhibits regulatory interactions with pathological processes such as inflammation and oxidative stress [55, 56]. Although ferroptosis is a relatively new and recently discovered type of cell death, it shows promising potential for attenuating EBI after SAH and may serve as a novel and effective target for SAH treatment.

Ferroptosis is a recently discovered form of cell death, and there is limited clinical research and translation focused on targeting iron-induced cell death for the treatment of SAH). Studies investigating the role of sirtuins in this context are even scarcer. Only one study conducted in the past two decades, utilizing a mouse model of SAH with crossed anterior pool injection, has demonstrated that activation of SIRT1 can inhibit iron-induced cell death by increasing the expression levels of GPX4 and FSP1 after SAH. This study elucidated, for the first time, the involvement of FSP1/CoQ10-mediated iron depletion in the development of EBI after SAH. Furthermore, the study revealed multiple mechanisms through which ferroptosis contributes to the pathogenesis of EBI after SAH, including upregulation of ASCL4 expression, increased expression of iron uptake proteins (e.g., TFR and DMT1) due to iron overload, reduced GPX4 expression, and inactivation of the FSP1-mediated antioxidant pathway [57]. Therefore, SIRT1 may represent a novel therapeutic target for inhibiting ferroptosis following SAH.

### Sirtuins and Neuroinflammation

During the early stages of subarachnoid hemorrhage (SAH), degradation products from erythrocytes rapidly accumulate in the subarachnoid space, triggering an inflammatory response that contributes to the progression of brain injury [58, 59]. Increasing evidence suggests that the neuroinflammatory response is a pervasive factor in both EBI and delayed brain injury (DBI) following SAH, playing a crucial role in the pathogenesis of EBI in particular [60]. The inflammatory cascade is primarily initiated by molecules released from extravasated blood and damaged brain tissue, accompanied by activation and infiltration of immune cells at the site of injury [61]. In addition to the classical neuroinflammation, SAH also leads to aseptic neurogenic inflammation, characterized by the release of potent vasoactive neuropeptides. This aseptic neurogenic inflammatory response is an independent predictor of mortality in SAH patients and can exacerbate classical neuroinflammation through a positive feedback loop involving inflammatory mediators [62–64].

The central nervous system (CNS), protected by the blood–brain barrier (BBB), has its own innate immune system, primarily composed of microglia, along with astrocytes and oligodendrocytes [65]. Following SAH, damage-associated molecular patterns (DAMPs) are released by neurons, astrocytes, microglia, and endothelial cells due to tissue injury. DAMPs activate local and peripheral immune cells, leading to the release of inflammation-associated proteins and cytokines that fuel the inflammatory response [66]. Microglial activation occurs within minutes of SAH onset, resulting in rapid morphological changes characterized by enlarged cell bodies, shortened and thickened axons, and an amoeboid appearance [67, 68]. Activated microglia release various factors such as IL-1, IL-6, iNOS, TNF-α, NO, and MMP-9, which contribute to detrimental consequences including cerebral vasospasm, microthrombosis, blood–brain barrier disruption, and neuroapoptosis. This has a significant impact on disease regression and progression in patients [69]. Additionally, the transition of microglia from an anti-inflammatory phenotype (M2) to a pro-inflammatory phenotype (M1) plays a crucial role in microglial activation and the subsequent neuroinflammatory response. The M1 phenotype facilitates the release of pro-inflammatory cytokines that worsen nerve injury, while the M2 phenotype promotes the release of neurotrophic factors that contribute to nerve repair [70]. Similar to microglia, astrocytes have the ability to synthesize and secrete inflammatory factors, including cytokines and chemokines, which participate in the inflammatory process of SAH [71].

Apart from inflammatory cells, inflammation-associated proteins such as nuclear factor-kB (NF-kB), intercellular adhesion molecule-1 (ICAM-1), high mobility group box 1 (HMGB1), NLRP3 inflammasomes, toll-like receptors (TLRs), and mitogen-activated protein kinase (MAPK) also play critical roles in post-SAH inflammation [72]. Furthermore, pro-inflammatory cytokines like interleukin-1β (IL-1β), interleukin-6 (IL-6), and tumor necrosis factor-α (TNF-α) upregulate specific cell adhesion molecules on brain capillary endothelial cells, directly damage peripheral nerve cells, increase leukocyte recruitment, induce neuroinflammation, degrade the brain capillary endothelial basement membrane, leading to BBB disruption, and contribute to various apoptotic cell death processes, further exacerbating brain injury after SAH [73].

Therefore, modulating the neuroinflammatory response may alleviate early EBI following SAH and potentially improve patient prognosis.

Sirtuins have a regulatory role in the inflammatory response of the CNS. Numerous studies have reported a close association between SIRT1 and neuroinflammation, with NF-kB, p53, Nrf2, FOXO, and HIF identified as substrates for SIRT1 deacetylation, both in histone and non-histone proteins [74]. Interestingly, levels of SIRT1 protein were found to be significantly elevated in the early stages of SAH, peaking at 24 h after the event. The endogenous SIRT1-regulated p53 pathway reduces p53 acetylation by inhibiting FOXO1 and NF-кB. Activation of the SIRT1 pathway after SAH leads to a significant reduction in levels of IL-1b, IL-6, and TNF-a, decreased activation of microglia, reduced levels of Bax and cleaved-caspase-3, increased expression of Bcl-2, and improved neuroinflammation following SAH [75–77]. SIRT1 has also been found to be closely associated with neuroinflammation in multiple studies. TLR4, highly expressed in microglia, can induce the production of various pro-inflammatory cytokines and chemokines, including IL-1b, IL-6, TNF-a, and ICAM-1. Astaxanthin (ATX) effectively alleviates brain inflammation by activating SIRT1 and inhibiting the TLR4 signaling pathway. However, this effect is ineffective in mice lacking the TLR4 gene [78]. Pinocembrin upregulates SIRT1 expression, suppresses NF-кB and microglia activation, and ameliorates inflammation following EBI [79]. In EBI after SAH, resveratrol (RSV) inhibits pro-inflammatory cytokines (IL-1b, IL-6, and TNF-a) through the AMPK/SIRT1 cascade [80]. Furthermore, melatonin has been shown to downregulate Ac-NF-kB and Bax expression while upregulating SIRT1 expression, indicating that melatonin attenuates neuroinflammation after SAH partly through the melatonin receptor (MR)/SIRT1/NF-kB signaling pathway [81]. Phosphodiesterase-4 (PDE-4) plays a crucial role in various CNS injuries. As a PDE4 inhibitor, rolipram significantly increases the expression of SIRT1, leading to the inhibition of NF-kB activation in EBI after SAH. Rolipram ameliorates inflammation by upregulating the expression of the protective cytokine IL-10, inhibiting the expression of pro-inflammatory cytokines TNFa, IL-1ß, and IL-6, and suppressing microglial activation [82].

Additionally, intense brain inflammation following SAH is associated with the substantial activation of the HMGB1/NF-kB pathway [83, 84]. Growing evidence suggests that SIRT1 regulates HMGB1 hyperacetylation and inhibits HMGB1 translocation release. Increased expression of SIRT1 inhibits HMGB1/NF-KB activation-mediated inflammatory responses after SAH. The selective SIRT1 inhibitor EX527 reverses the SIRT1 activation induced by cycloastragenol (CAG) and attenuates the anti-inflammatory and neuroprotective effects of CAG on SAH, as evidenced by the upregulation of TNF-a, IL-1b, IL-6, ICAM-1 release, and microglial activation [76].

Oleanolic acid (OA) reduces the acetylation level of HMGB1 by increasing the expression of SIRT1, rather than inhibiting the JAK/STAT3 pathway. This promotion of HMGB1 deacetylation inhibits its translocation from the nucleus to the cytoplasm, thus reducing the serum level of HMGB1. OA exerts its anti-inflammatory effects through the SIRT1 signaling pathway, downregulating the expression of TLR4, TNF-a, IL-1b, and NF-kB, thereby exerting anti-inflammatory effects. Furthermore, it has been observed that HMGB1 is predominantly expressed in neurons and associated with apoptosis in EBI after SAH, while in DBI after SAH, it is primarily expressed in microglia and associated with immune activation. This finding remains controversial but represents a valuable issue that merits further investigation [85].

Furthermore, NLRP3 inflammasomes, functioning as multiprotein oligomers, play a pivotal role in activating the inflammatory response, promoting IL-1b maturation, and inducing IL-1b release, ultimately resulting in inflammation and tissue damage [86]. Activation of SIRT1 has been found to reduce the acetylation of FoxO1 and P53 and inhibit NLRP3 inflammasome activation [87]. Another study demonstrated that SIRT1 could alleviate EBI after SAH by modulating NLRP3 inflammasome signaling to shift microglial phenotype from M1 to M2 phenotype [88]. Recently, in an estrogen-deficient aneurysm model, the deficiency of estrogen receptor alpha (ERα) and SIRT1 may contribute to inflammation and tissue damage, promoting the activation of the NLRP3/IL-1b/MMP-9 pathway, thereby increasing the risk of intracranial aneurysm rupture and SAH [89]. Experimental evidence also suggests the involvement of other members of the SIRT family in neuroinflammation after SAH. For instance, bexarotene activates retinoid X receptors (RXR) to reduce neuroinflammation after SAH through the PPARγ/SIRT6/FoxO3a pathway [90].

Neuroinflammation is a common cause of brain injury in SAH, and while some studies suggest that targeting neuroinflammation could be a therapeutic option, other clinical studies have found that modulating inflammation after SAH does not yield beneficial effects [91]. Acute neuroinflammation is often considered a protective response, whereas chronic neuroinflammation is deemed harmful and damaging to neural tissue. It is important to recognize that inflammatory pathways and mediators may have both protective and detrimental roles at different stages [92]. Therefore, the management of neuroinflammation after SAH should not solely focus on suppressing inflammation; it should also consider avoiding the blockade of neuroprotective immune responses. In the future, individualized treatments tailored to the timing and intensity of the inflammatory response in each patient should be developed. Modulation of SIRTs presents a promising avenue for achieving this goal.

### Sirtuins and Oxidative Stress

Oxidative stress (OS) is a crucial factor contributing to early brain injury (EBI) after subarachnoid hemorrhage (SAH). The brain is highly vulnerable to oxidative damage due to its abundance of polyunsaturated fatty acids in the tissue. Following SAH, an excessive generation of free radicals occurs, depleting the endogenous antioxidant system and leading to the downregulation of key antioxidant enzymes such as superoxide dismutase (SOD), glutathione peroxidase, and catalase in brain tissue, thereby disrupting redox homeostasis. Additionally, SAH-induced CVS and cerebral edema result in cerebral ischemia, which leads to the production of substantial amounts of reactive oxygen species (ROS) such as oxygen ions (O2-) and hydrogen peroxide (H2O2) [93, 94]. The release of oxyhemoglobin or hemoglobin from erythrocytes following erythrocyte lysis after SAH further exacerbates the situation as these molecules readily undergo oxidation to form methemoglobin [95]. High levels of Fe2 + and Fe3 + can react with H2O2 and O2- through the Fenton reaction, generating hydroxyl radicals, the most toxic ROS. Hydroxyl radicals directly damage neurovascular units, leading to neurological impairment, and the remaining ROS contribute to mitochondrial dysfunction, perpetuating a detrimental cycle of ROS production. Moreover, oxidative stress induced by free radicals results in lipid peroxidation, protein degradation, and DNA damage, ultimately leading to neuronal apoptosis, endothelial cell damage, and BBB disruption [96, 97]. Importantly, a growing body of evidence suggests that the prevention of excessive ROS can alleviate EBI after SAH [98, 99]. Therefore, targeting the prevention of oxidative damage may represent a promising therapeutic approach to improve the prognosis following subarachnoid hemorrhage.

Despite the substantial evidence of oxidative stress in SAH, the use of antioxidants as a treatment modality for EBI in SAH is not recommended in the definitive guidelines [100, 101]. Nevertheless, numerous studies have highlighted the regulatory role of SIRTs in various biological processes, with SIRT1 particularly known for its antioxidant properties. SIRT1 deacetylation has been shown to inhibit oxidative pathways mediated by FoxOs, NF-кB, and to some extent, P53, offering neuroprotection against EBI in animal models. Additionally, a decrease in SIRT1 levels was associated with exacerbated cortical oxidative damage, microglial activation, and the release of pro-inflammatory cytokines [75, 76]. Another antioxidant, ATX, exerts its protective effects against SAH-induced oxidative stress by increasing SIRT1 expression and inhibiting the TLR4 signaling pathway [78]. Fx, derived from seaweed, attenuates SAH-induced reactive oxygen species (ROS) production and lipid peroxidation through a SIRT1-dependent pathway. Fx inhibits the acetylation of downstream substrates FoxO1 and p53, restores the activity of endogenous antioxidant enzymes, blocks mitochondrial cytochrome c release, and restores mitochondrial morphology following SAH [41].

Activation of the melanocortin 1 receptor by BMS-470539 inhibits oxidative stress and mitochondrial division after EBI through the AMPK/SIRT1/PGC-1α signaling pathway [40]. Nrf2, a basic leucine zipper protein (bZIP), plays a crucial role in maintaining cellular redox homeostasis. Activation of Nrf2 induces the expression of several antioxidant genes, including glutathione peroxidase (GSH-Px), superoxide dismutase (SOD), and heme oxygenase-1 (HO-1) [102]. The Keap1/Nrf2/ARE pathway has been identified as an antioxidant target in models of the oxidative stress response after SAH [103]. Under the regulation of SIRT1 activation, salvianolic acid B ameliorates oxidative damage by promoting Nrf2 nuclear translocation. Knockdown of Nrf2 significantly reverses the antioxidant effect of salvianolic acid B, indicating a positive feedback loop between SIRT1 and Nrf2 signaling [104]. Two animal studies have shown that isoglycyrrhizin (ISL) and the resveratrol (RVS) analog pterostilbene (PTE) can mitigate severe oxidative damage by activating the SIRT1/Nrf2 pathway and enhancing the activity of endogenous antioxidant enzymes [105, 106]. Recently, other members of the SIRT family, particularly SIRT3, have gained attention for their antioxidant mechanisms in various neurological disorders, including ischemic stroke, Huntington's disease, and Alzheimer's disease [107]. SIRT3 is significantly activated in animal models of SAH, both in vivo and in vitro. The transcriptional coactivator peroxisome proliferator-activated receptor γ coactivator 1-α (PGC-1α) is involved in regulating the antioxidant activity of SIRT3 after SAH, thereby enhancing the endogenous antioxidant response [108]. In a time-dependent manner, the expression of SIRT3 in cortical neurons was found to decrease, and both mRNA and protein expression of SOD2 showed a positive correlation with SIRT3 expression [109]. Furthermore, melatonin was shown to reduce ROS levels, inhibit the expression of SOD2 and the lipid peroxidation marker malondialdehyde (MDA), and regulate SIRT3 expression [110]. SIRT6 has demonstrated protective effects against cardiac I/R injury by upregulating antioxidants and suppressing OS. Expanding on this success, activation of the RXR could potentially ameliorate certain neurological deficits after SAH by modulating the PPARγ/SIRT6/FOXO3a pathway [90]. Currently, four free radical scavengers, including edaravone, tirazadex mesylate, nicardipine, and ebselenolide, are undergoing clinical trials, but unfortunately, no neuroprotective effects have been reported [93]. The modulation of SIRTs to reduce oxidative stress after SAH holds promise as a new avenue of investigation that merits further exploration.

### Sirtuins and Apoptosis Versus Autophagy

Apoptosis is a prominent pathological process in EBI, and experimental studies have demonstrated its close association with brain injury following SAH [111]. Apoptosis is characterized by distinct morphological changes such as cell shrinkage, nuclear fragmentation, chromatin condensation, and chromosomal DNA fragmentation [112]. Neuronal apoptosis has been observed within 10 min of SAH onset and continues for up to 24 h [113]. Due to the limited regenerative capacity of the human brain, EBI-induced neuronal injury can lead to permanent damage and long-term neurological impairment [114]. Apoptosis can occur through three different pathways: the extrinsic pathway, the intrinsic pathway, and the endoplasmic reticulum stress-induced pathway, depending on the site of apoptosis. Molecular mechanisms such as p53 and oxidative stress pathways are also implicated in SAH-induced apoptosis [115]. The extrinsic pathway, also known as the death receptor pathway, involves pro-apoptotic death receptors such as TNFR1, TNFR2, Fas, and TRAIL-R1 (DR 4) and TRAIL-R2 (DR 5) [116]. The upregulation of Fas and TNF ligands following SAH binds to death receptors, activating the caspase cascade [117, 118]. The intrinsic pathway, known as the mitochondrial pathway, is primarily regulated by the B-cell lymphoma-2 (Bcl-2) family of proteins. Upon apoptotic stimulation during SAH, mitochondrial outer membrane permeability increases, and cytochrome C is released from the mitochondria into the cytoplasm, where it forms apoptotic complexes with apoptotic protease activator 1 (Apaf-1), leading to the activation of caspase-9. Caspase-3 is subsequently activated, triggering apoptosis [119, 120]. Endoplasmic reticulum stress (ERS) occurs due to imbalanced ion levels and the accumulation of misfolded or unfolded proteins in the endoplasmic reticulum [121]. ERS disrupts calcium homeostasis and affects mitochondrial and Bcl-2 family protein activities, resulting in apoptosis. It also activates the cysteine protease cascade, further influencing apoptosis [122, 123]. Recent studies have revealed morphological changes in the endoplasmic reticulum within 6 h of SAH, with cortical neuron swelling peaking at 24 h and subsiding within 24–48 h [124]. These pathways interact with each other and contribute to the initiation and regulation of apoptosis after SAH. By targeting these pathways, it may be possible to effectively alleviate apoptosis, thereby mitigating nerve injury and promoting neurological recovery after SAH.

Autophagy is a cellular repair process that maintains intracellular homeostasis by selectively degrading and recycling cytoplasmic components and eliminating unwanted cellular entities [125]. Following SAH, the autophagic pathway is activated and reaches its peak at 24 h [22]. Activation of autophagy has been shown to exert neuroprotective effects [126]. In a rat model of intravascular perforation, autophagy activation reduced the translocation of Bax from the cytosol to the mitochondrial membrane, thereby counteracting apoptotic effects [127]. The mitochondrial pathway is believed to be involved in autophagy-mediated regulation of apoptosis in EBI after SAH [128]. Studies have consistently demonstrated that proper regulation of autophagic mechanisms has a pro-survival effect and reduces apoptotic cell death after SAH. However, when SAH surpasses a certain stress threshold, autophagic mechanisms can contribute to increased apoptotic cell death [129–132]. Besides autophagy and apoptosis, necrosis may also occur simultaneously in neurons after SAH, resulting in a mixed pattern of cell death morphology. Moreover, extensive crosstalk exists between autophagic and apoptotic pathways [133]. Recent research has highlighted the importance of reducing neuronal death to ameliorate neurological dysfunction and improve patient prognosis [134, 135]. Therefore, future studies should focus on understanding the interplay between autophagy, apoptosis, and necrosis rather than studying them in isolation. A comprehensive understanding of the relationship among these pathways holds promise for advancing SAH treatment options.

Several studies have demonstrated the neuroprotective effect of sirtuins on apoptosis in EBI cells after subarachnoid hemorrhage and their involvement in autophagy regulation. Numerous studies focusing on SIRT1 have revealed its ability to ameliorate apoptosis after SAH through multiple pathways. For instance, wogonoside and resveratrol can activate SIRT1, deacetylate p53, inhibit p53-mediated transcriptional activity, increase cleaved caspase-3 and Bax levels, and increase Bcl-2 levels, thereby blocking apoptosis [76, 136, 137]. Pterostilbene (PTE) effectively upregulates SIRT1 expression, promotes nuclear Nrf2 accumulation, inhibits microglial activation and pro-inflammatory mediators after SAH, and attenuates oxidative damage and neuroinflammation, directly inhibiting apoptosis [106]. Magnesium lithospermate B (MLB) and melatonin (Mel), extracted from Salvia miltiorrhiza, induce upregulation of Bcl-2 expression and downregulation of Bax expression through the SIRT1/NF-κB pathway, exerting anti-apoptotic effects and inhibiting apoptosis after SAH [77, 81]. Moreover, inhibition of phosphodiesterase-4 (PDE4), an enzyme that hydrolyzes cAMP, significantly increases SIRT1 expression after SAH, upregulates Akt phosphorylation, and decreases the expression of apoptotic proteins, thereby inhibiting neuronal apoptosis after SAH [138]. Other members of the sirtuin family, such as SIRT3, have also been implicated in the regulation of apoptosis after SAH in animal studies. Puerarin (Pur) reduces cortical neuronal degeneration and apoptosis by downregulating the Bcl-2/Bax/cleaved caspase-3 apoptotic pathway and upregulating the SIRT3/SOD2 antiapoptotic pathway [139]. Tauroursodeoxycholic acid (TUDCA) attenuates apoptosis after SAH by activating the TGR5/SIRT3 pathway, leading to the inhibition of apoptotic protein expression. Conversely, knockdown of TGR5 by siRNA abolishes the beneficial effects of TUDCA [140]. Unfortunately, the role of SIRTs in autophagy after SAH remains poorly studied. However, resveratrol has been shown to mediate autophagy and apoptosis in SAH through the regulation of the Akt/mTOR pathway. Resveratrol stimulates autophagy through the AMPK/SIRT1 signaling pathway, inhibiting the release of pro-inflammatory cytokines and neuronal apoptosis, thereby uncovering a novel molecular mechanism for its protective effect in subarachnoid hemorrhage [80]. Autophagy plays a crucial role in maintaining intracellular homeostasis in the brain following SAH, exerting a pro-survival effect and reducing apoptotic cell death. Thus, modulating autophagy and the crosstalk with apoptosis may hold therapeutic benefits in the context of SAH. Additionally, sirtuins can regulate both apoptosis and autophagy. The relationship between apoptosis and autophagy in SAH presents an intriguing target for further research and therapeutic interventions.

### Sirtuins and Blood–Brain Barrier Disruption

The blood–brain barrier primarily consists of capillary endothelial cells, pericytes, astrocytes, and the vascular basement membrane. Capillary endothelial cells are closely connected, resulting in minimal cell gaps. In normal physiological conditions, the BBB prevents most substances, except for a few lipid-soluble molecules and gases, from crossing into the brain, including plasma components and red blood cells [141, 142]. However, under pathological conditions, the BBB becomes compromised, allowing harmful blood components such as thrombin and fibrinogen to enter the brain parenchyma and directly expose the brain tissue to these toxic substances [143]. Increased BBB permeability also facilitates the infiltration and restricted migration of immune cells, like leukocytes, into the brain parenchyma, leading to the release of various cytokines, chemokines, reactive oxygen species, and proteases. This further exacerbates brain tissue damage, raises intracranial pressure, triggers neuronal apoptosis, and can contribute to epilepsy. Additionally, BBB permeability disruption can cause dysfunction in endothelial cells and the breakdown of tight junctions, resulting in the formation of cerebral edema [144–146].

Brain edema following SAH is primarily categorized as vasogenic and cytotoxic. Vasogenic edema occurs due to the leakage of plasma proteins and fluid accumulation in the brain interstitium [147]. On the other hand, cytotoxic edema is a consequence of reduced cerebral blood flow (CBF) resulting from increased intracranial pressure (ICP) after SAH. This reduction in blood flow leads to whole-brain ischemia, ATP depletion, and loss of energy from key "pumps" like Na + -K + -ATPase and Ca2 + -ATPase, eventually causing cellular swelling [148, 149]. Studies have shown that BBB disruption occurs soon after SAH onset, even before observable changes on MRI. Researchers have provided evidence that BBB damage begins as early as 30 min after SAH, peaks at 3 h, and can be assessed at 72 h by evaluating tight junction proteins such as occludin and ZO-1. Clinical data also indicate that approximately 8% of patients present with whole-brain edema upon admission using cranial CT, and an additional 12% develop significant brain edema within 6 days of SAH [12, 150]. Severe cerebral edema often leads to elevated ICP, acute cerebral ischemia, brain herniation, and potentially fatal outcomes for patients. Therefore, it is crucial to safeguard the integrity of the blood–brain barrier, reduce the occurrence of cerebral edema, and improve the prognosis of individuals with SAH [151].

Hypertonic saline, hyperosmolar agents, therapeutic hypothermia, barbiturates, non-peptide antidiuretic hormone receptor antagonists, calcium channel blockers, and decompression with debridement are commonly utilized in clinical practice to mitigate cerebral edema and manage intracranial pressure. However, the precise efficacy of these interventions has not been adequately evaluated or retrospectively analyzed [152–155]. Cerebral edema, which results from blood–brain barrier dysfunction, is a significant independent risk factor for the high morbidity and mortality associated with subarachnoid hemorrhage [156]. Maintaining the integrity of the blood–brain barrier relies on tight junction proteins, including claudin-5, occludin, and ZO-1, which are crucial components of the blood–brain barrier structure and regulation [157–159]. Recent experimental studies have demonstrated that astragaloside, resveratrol, and MEL, as SIRT1 activators, enhance SIRT1 expression and suppress p53 activation through deacetylation. Additionally, they downregulate the activity and expression of matrix metalloproteinase-9 (MMP-9) while increasing the expression of ZO-1, claudin-5, and occludin. These actions contribute to protecting the blood–brain barrier and ameliorating brain edema. Notably, the administration of the potent SIRT1 inhibitor sirtinol (SIR) hampers SIRT1 activation, reverses the aforementioned protein expression and outcomes, and exacerbates brain edema following experimental subarachnoid hemorrhage [136, 137, 160]. Modulating SIRT1 may therefore represent a promising therapeutic target for effectively reducing brain edema subsequent to subarachnoid hemorrhage.

## Conclusion and Outlook

SAH represents a fatal type of stroke, and even those patients who survive often face a grim prognosis, including conditions like hemiplegia, aphasia, cognitive dysfunction, or even death. These outcomes significantly impact patients' quality of life and pose a substantial socioeconomic burden [1, 161, 162]. Experimental studies have highlighted the intricate nature of early EBI after SAH, which arises from a combination of mechanisms rather than a singular cause. These mechanisms can exhibit synergistic effects and temporal variability. However, most animal model studies exploring the role and mechanisms of sirtuins in regulating SAH have focused solely on one aspect, neglecting the investigation of other potential mechanisms underlying EBI. Additionally, there remains controversy regarding the expression of SIRT1 in astrocytes. Despite extensive research into the mechanisms of EBI, the exact molecular pathway remains unclear, impeding the development of effective and targeted therapeutic interventions. Sirtuins, as epigenetic regulators, participate in numerous physiological activities such as genome stabilization, cancer, stress response, apoptosis, metabolism, aging, proliferation, and inflammation [163–165]. Generally, sirtuins confer neuroprotective effects on CNS cells, with a possible synergistic enhancement of intracerebral homeostasis through distinct cellular regulatory pathways involving SIRT1-SIRT7 proteins. Among the sirtuins, SIRT1 was the first to be identified and extensively studied for its involvement in various disease-modulating mechanisms [166]. Presently, studies examining sirtuins in the context of SAH are primarily limited to animal and cellular models, with a scarcity of clinical evidence. Although animal models, including endovascular perforation and autologous blood injection, are widely utilized to mimic SAH [167], more comprehensive investigations are required to establish a solid understanding of the role of sirtuins in SAH pathophysiology.

Regrettably, the intricate physiological conditions associated with human SAH are difficult to fully replicate in animal models. Consequently, it is crucial to identify a more suitable SAH model for future investigations. Moreover, extensive clinical studies involving humans are necessary to confirm the mechanisms of action, efficacy, and safety of potential therapies. Sirtuins have garnered significant attention as promising therapeutic targets, leading to the development of specific sirtuin activators and inhibitors. These compounds serve as valuable tools to study sirtuin activity and hold potential as therapies for associated diseases. Clinical trials utilizing sirtuin activators have been conducted to prevent age-related conditions [168, 178]. Furthermore, many sirtuin modulators, including substances like resveratrol, melatonin, and tannic acid, are derived from natural sources, boasting advantages such as environmental friendliness, accessibility, and minimal side effects [169, 170]. Additionally, approximately one-third of SAH patients experience DCI occurring between days 4 and 14 or even later following EBI [171, 172]. Recently, a new perspective has emerged challenging the conventional classification of SAH stages, suggesting that DCI is a continuation of EBI rather than a distinct phase [173]. Intriguingly, a significant body of research has demonstrated the protective effects of enhanced SIRT1 expression in DCI after SAH [174–177]. However, translating the findings of experimental studies on sirtuins into clinical trials poses several challenges. These include the complex and bidirectional nature of sirtuin activity, genetic and biochemical differences between human diseases and animal models, and the difficulty of identifying suitable candidate molecules with favorable pharmacodynamic profiles and bioavailability [178]. Nevertheless, the field of sirtuin modulation has emerged as a promising avenue for drug development. Its potential in treating brain injury and improving prognosis following SAH presents an exciting direction for future research, warranting further exploration despite the associated risks and challenges.
